# Building a virtual community of practice **–** The evolution of the TBPPM learning network, India chapter

**DOI:** 10.1016/j.jctube.2024.100419

**Published:** 2024-02-15

**Authors:** Vijayashree Yellappa, Kusum Moray, Petra Heitkamp, Joel Shyam Klinton

**Affiliations:** a TBPPM Learning Network - India Chapter, India; b TBPPM Learning Network, Montreal, Canada; c McGill International TB Centre, Montreal, Canada

**Keywords:** Tuberculosis, PPM, Community of practice, Learning network, Private sector

## Abstract

India's National Tuberculosis (TB) Elimination Program strategically involves private providers to achieve its 2025 goal of ending TB. The government's patient-provider support agency scheme (PPSA) aims to expand the Public-Private Mix (PPM) strategy using domestic resources, though challenges persist in cross-learning and documentation. The TB Public Private Mix Learning Network (TBPPM-LN) launched its India chapter in 2021, connecting PPM stakeholders virtually. With 600 + members, TBPPM-India, acting as a digital Community of Practice, is pivotal in fostering a learning culture, leading knowledge-sharing initiatives, and disseminating TBPPM field innovations, contributing significantly to India's intensified efforts against TB.

India’s National Tuberculosis (TB) Elimination Program (NTEP) is striving to end TB by 2025. Despite the progress in TB control, India is far from achieving this goal due to several challenges [1]. One such challenge that needs urgent attention to end TB is the engagement of the vast and heterogeneous private health sector in the country [2]. The Government of India has been strategically engaging private providers in TB care and management through WHO recommended Public-Private Mix (PPM) approach [3]. A series of initiatives to create an enabling environment for the engagement of the private sector such as mandatory TB notification [2], a digital data management platform (Nikshay) [3], and a direct benefit transfer scheme for nutritional support [4] are in place.

A significant commitment from the Government is the rolling out of the patient-provider support agency scheme (PPSA) [5] in 2020, wherein domestic resources are mobilized to expand the PPM strategy. The scheme is promising; however, there is limited cross-learning and documentation of PPM innovations in the nation. Within this context, the TB Public Private Mix Learning Network (TBPPM-LN)’s India chapter was established in 2021 as an offshoot of the global TBPPM-LN, whose secretariat is hosted at McGill University, Canada [6]. TBPPM-LN is a virtual Community of Practitioners (CoP) of PPM stakeholders such as implementers, program managers, and academia. It facilitates the sharing of information, resources, and best practices in the domain, thus accelerating peer learning.

CoPs are characterized by a domain of knowledge, a community of people, shared practice, and understanding. A virtual CoP provides adaptable networking and knowledge management solutions that allow for cooperation, best practice exchange, and professional growth from the convenience of one's location. This bridges geographical boundaries and facilitates professional development, innovation, and communication. CoP is viewed as a set of nodes and links that can be utilized for learning, information flows, helpful linkages, joint problem-solving, and knowledge creation [7]. TBPPM-LN imbibes these characteristics of a CoP as a virtual network. In the two years (2021–2023) of its functioning, TBPPM-LN-India has stepped into the PPM ecosystem and has facilitated the cementing of a health systems ‘software’ with a focus on relationships, shared values, interests, and experiences. It continues to create a neutral platform for the PPM stakeholders, particularly the PPM implementers, and thus provides concrete supporting tools and activities.

To begin with, the TBPPM-LN India chapter started with a scoping exercise to understand the PPM landscape. An advisory group was formed, and key stakeholders were engaged through the creation of *targeted learning opportunities* and dialogue in the form of *webinars.* Of particular interest, were the closed meetings with PPSA implementers which brought out actionable insights. In response, the TBPPM LN India team created a *geo-tagged inventory of non-governmental organizations* eligible to participate in the PPSA scheme and continues to encourage NGOs to apply for PPSA schemes through competitive bidding. While interacting with PPM implementers, some organizations were found to be more experienced in PPM activities, while others requested guidance on the same. Such organizations were matched to create potential mentor–mentee tie-ups. PPSA organizations voiced a need to document their best practices and requested support in data analysis. To address this need, the TBPPM LN India signed a Memorandum of Understanding with The Indian Association for Preventive and Social Medicine, to support the PPM implementers with documentation. A *digital resource of relevant TBPPM material* is made available to the members on the platform’s website. A *compendium of the best TBPPM practices* [8] and *videos of virtual TB PPM field visits* [9] were launched at the UNION Conference 2023. The virtual field visits act as a promotional tool for PPM and provide a rich learning resource for various TBPPM stakeholders. With these initiatives, the TBPPM LN India has been acting as a knowledge broker, connecting practice and evidence.

The abovementioned tools and activities have been received well by the TBPPM stakeholders in the country and the utility of the platform has been recognized. Partners from diverse backgrounds have stated how the platform is bringing together TBPPM stakeholders, hence improving cross-learning and engagement. One of the PPM Implementers quotes, *“The India chapter is a platform with varied stakeholders- a good place to learn from each other in a big country like India, in different stages of progress.”* The TBPPM LN India chapter has developed a monitoring and evaluation framework with outcome indicators that align with the larger picture of the National Strategic Plan, as well as the focus areas of the global team. However, direct causation and impact are complex to establish in such learning network initiatives.

Wenger et al conceptualized a framework to explain how CoPs create value [10]. This framework was appropriate to showcase how far the TBPPM-LN India chapter has come, and the direction it will take in the future. Fig. 1 provides clarity on the value creation cycles and the alignment of TBPPM LN India activities with this framework. TBPPM-LN India is transitioning from cycle 3 of ‘applied value’ through promising practices to cycle 4 of ‘realized value’ of return on investment. The framework shows how intangible, qualitative aspects can drive the aspired impact of a culture shift. The shift is anticipated to be in terms of peer interactions across the country and the world to center on increasing the quality of care for patients with TB. As of 2024, the core team has grown, and the network is institutionalized by establishing a national secretariat at the Institute of Public Health, a research institute in India [11]. Looking forward, TBPPM LN India would continue its journey of brokering strategic partnerships and facilitating crucial cross-learning to address gaps like lack of visibility on the various TBPPM mechanisms in place.

**Fig. 1:**
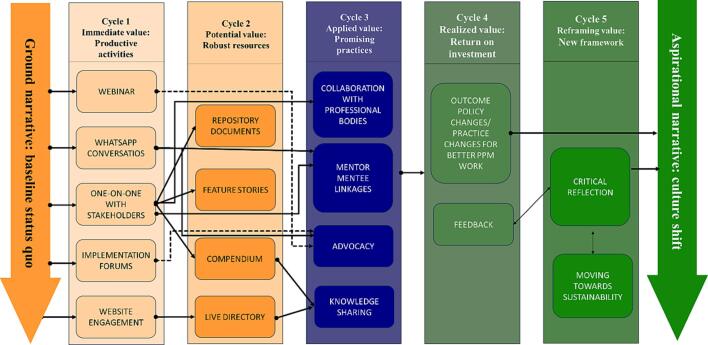
Value creation framework applied to TBPPM LN India chapter’s journey.

TBPPM-India has achieved a vast membership of 600 + members, and other tangible gains. The various stakeholders in the ecosystem including the advisory board have been supportive and are invested in the progress of TBPPM-LN India. Overall, a digital CoP such as TBPPM-LN India, acting as a ‘***health systems software’*** adds value to the status quo. In this crucial time, where India is accelerating its journey to end TB, TBPPM-LN India will continue to play an important role in supporting the overall learning culture, lead knowledge-sharing initiatives as a convenor, act as an orchestrator of dialogue and disseminate TBPPM field innovations by funneling learning in the appropriate direction.

## Funding

This work was supported, in whole or in part, by the Bill & Melinda Gates Foundation [INV-042531]. Under the grant conditions of the Foundation, a Creative Commons Attribution 4.0 Generic License has already been assigned to the Author Accepted Manuscript version that might arise from this submission.

## Ethical statement

This is an editorial and did not involve any participation from human or animal subjects.

## CRediT authorship contribution statement

Vijayashree Yellappa and Kusum Moray contributed equally as first authors. **Vijayashree Yellappa:** Conceptualization, Data curation, Writing – original draft. **Kusum Moray:** Conceptualization, Data curation, Writing – original draft. **Petra Heitkamp:** Writing – review & editing. **Joel Shyam Klinton:** Writing – review & editing.

## Declaration of competing interest

The authors declare that they have no known competing financial interests or personal relationships that could have appeared to influence the work reported in this paper.

## References

[1] The End TB Strategy. Accessed: Jan. 24, 2023. [Online]. Available: https://www.who.int/teams/global-tuberculosis-programme/the-end-tb-strategy.

[2] TB Notification Govt Order dated 07 05 2012.pdf. Accessed: Nov. 20, 2023. [Online]. Available: https://tbcindia.gov.in/WriteReadData/l892s/8249592141TB%20Notification%20Govt%20%20Order%20dated%2007%2005%202012.pdf.

[3] Nikshay. Accessed: Nov. 20, 2023. [Online]. Available: https://www.nikshay.in/.

[4] Direct Benefit Transfer :: Central TB Division. Accessed: Nov. 20, 2023. [Online]. Available: https://tbcindia.gov.in/index1.php?lang=1&level=1&sublinkid=4802&lid=3316.

[5] Operations Manual for Partnerships_10-04-23.pdf. Accessed: Feb. 01, 2024. [Online]. Available: https://tbcindia.gov.in/WriteReadData/l892s/3641774273Final%20Operations%20Manual%20for%20Partnerships_10-04-23.pdf.

[6] TBPPM Learning Network. Accessed: Jan. 08, 2024. [Online]. Available: https://www.tbppm.org/.

[7] Building Community : A Primer - 2018 update. Accessed: Jan. 25, 2023. [Online]. Available: https://openknowledge.worldbank.org/handle/10986/34014.

[8] Compendium of Public Private Mix for Tuberculosis Control in India Accelerating Efforts to End Tuberculosis. 2023.

[9] Virtual PPM Field Visits - India, (Nov. 17, 2023). Accessed: Feb. 01, 2024. [Online Video]. Available: https://www.youtube.com/watch?v=LsSaJQpFoZc.

[10] Wenger E., Trayner B., de Laat M. (2011).

[11] Institute of Public Health, Bengaluru | IPH | Academic Institute. Accessed: Jan. 24, 2024. [Online]. Available: https://iphindia.org/.

